# Shikonin Inhibits Der p 2-Induced Cytokine and Chemokine Expression in Dendritic Cells in Patients with Atopic Dermatitis

**DOI:** 10.1155/2020/9506363

**Published:** 2020-07-31

**Authors:** Chung-Yang Yen, Wen-Dee Chiang, Shang-Yong Liu, Sheng-Jie Yu, Ching-Liang Hsieh

**Affiliations:** ^1^Department of Dermatology, Taichung Veterans General Hospital, Taichung 40705, Taiwan; ^2^Department of Food Science, Tunghai University, Taichung 40704, Taiwan; ^3^Department of Education and Research, Kaohsiung Veterans General Hospital, Kaohsiung 81300, Taiwan; ^4^Chinese Medicine Research Center, China Medical University, Taichung 40402, Taiwan; ^5^Graduate Institute of Acupuncture Science, College of Chinese Medicine, China Medical University, Taichung 40402, Taiwan; ^6^Department of Chinese Medicine, China Medical University Hospital, Taichung 40447, Taiwan

## Abstract

Atopic dermatitis (AD) is a common inflammatory skin disorder. Shikonin, the active component of *Lithospermum erythrorhizon* extract, exhibits anti-inflammatory effects. The objective of the present study was to investigate the effect of shikonin on proinflammatory cytokines and chemokine in patients with AD. Ten patients with AD who were allergic to house dust mite (HDM) and seven healthy controls were recruited in this study. Peripheral blood mononuclear cells were isolated, and CD14^+^ cells were further selected and differentiated to dendritic cells. Dendritic cells stimulated using Der p 2, the major HDM allergen, were cotreated with shikonin for 24 hours, and dexamethasone was used as a control. Culture supernatants were collected, and proinflammatory cytokine and chemokine concentrations were analyzed using a multiplex assay system. Shikonin significantly inhibited Der p 2-induced expression of interleukin (IL)-6, IL-9, and IL-17A; monocyte chemoattractant protein (MCP)-1; macrophage inflammatory protein (MIP)-1*α*; MIP-1*β*; and Chemokine (C-C motif) ligand 5 (RANTES). The inhibitory effects of shikonin on IL-9, MIP-1*β*, and RANTES expression were stronger than those of dexamethasone. Therefore, Shikonin can be considered a promising drug for AD treatment because it inhibits different inflammatory cytokines expression.

## 1. Introduction

Atopic dermatitis (AD) is usually a lifelong disorder and requires long-term treatment. AD is a chronic, complex, and often relapsing inflammatory skin disorder. Pruritus, xerosis, and eczematous lesions are often observed in patients with AD [1]. The prevalence of AD has been reported to vary from 0.3% to 6.2% in Europe, 3.2% to 10.7% in the United States, and 2.9% in Japan [2–4]. Itching and pain disrupt daily activities and sleep in patients with AD, which adversely affect their quality of life. Furthermore, mood changes, social isolation, and depression were reported to affect patients with moderate to severe AD [5]. High risks of comorbidities, such as malignancies, autoimmune diseases, cardiovascular diseases, and neuropsychiatric diseases, and an increase in economic burden, are correlated with the severity of AD [6]. In the United States, the estimated cost of treating AD was reported to be more than five billion dollars per year [7]. Hence, developing effective new drugs is a promising method to reduce the cost of treating AD.

Thus far, medications used for treating AD were mainly used to control itching, repair skin, and ameliorate skin inflammation and infection. Topical application of calcineurin inhibitors, steroids, and antibiotics, as well as light therapy, was used as first-line medication. Oral steroids and immunosuppressive drugs effectively reduced the symptoms of moderate to severe AD [8]. Recently, the use of biological agents against interleukin (IL)-4R*α* was reported to treat AD [9]. However, the long-term use of steroids and immunosuppressive drugs exhibited side effects, including thinning of the skin, increased systemic infection, osteoporosis, and potential cancer risk. Moreover, high costs caused poor treatment persistence, and poor treatment adherence contributed to the adverse effects of inadequate treatment [10]. Therefore, the development of alternative low-cost remedies with minimal side effects, which are suitable for long-term use, is necessary.

In the skin of patients with AD, the infiltration of immune cells, including dendritic cells, macrophages, Th2 cells, Th17 cells, and eosinophils, was observed. Crosstalk among these immune cells is reported to constitute the pathogenesis of AD. Among the immune cells, dendritic cells and macrophages were reported to play the most crucial role in initiating a Th2-cell immune response and skin inflammation [11]. Fc*ε*RI expressed on the surface of dendritic cells was reported to bind with IgE; after challenge with the allergen, the number of dendritic cells increases in the epidermis and dermis, and the dendritic cells produce proinflammatory cytokines to enhance inflammation and differentiation of Th2 cells [12]. In AD, macrophages accumulate in the skin tissue and serve as antigen-presenting phagocytes against pathogens on the skin. During skin inflammation, macrophages produce proinflammatory cytokines and growth factors to enhance inflammatory responses [13]. Thus, inhibiting the activation of dendritic cells and macrophages has become the primary target for developing new drugs for treating AD.

**Figure 1:**
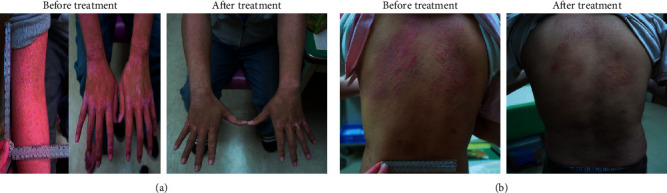
Effect of Tzu-Yun ointment (TYO) on patients with AD. (a) A 23-year-old man and (b) A 26-year-old man (left panel, before treatment) showed marked improvement in erythema and swelling after 8 weeks of treatment with topical application of TYO (right panel, after treatment).

In our previous report, we demonstrated that Tzu-Yun ointment (TYO) effectively reduced scores on the Eczema Area and Severity Index (EASI) and Three Item Severity in patients with AD after 8 weeks of treatment [14]. The major active component of TYO is shikonin, which is isolated from the root of a traditional Chinese herb, *Lithospermum erythrorhizon*. The topical application of TYO for 8 weeks effectively ameliorated dermatitis in patients with AD (Figure 1). Shikonin was reported to inhibit ovalbumin-induced activation of dendritic cells and attenuate allergic airway hyperresponsiveness in a murine model [15]. Furthermore, shikonin has been reported to reduce inflammation through antioxidation and inhibit chemotaxis in mononuclear macrophages and expression of genes related to lipopolysaccharide (LPS)-induced inflammation in macrophage cell lines [16–18]. These results suggest that shikonin exhibits multiple inhibitory effects on immune cells. However, information regarding the effects of shikonin on dendritic cells in patients with AD is not currently available.

In this study, we investigated the potential therapeutic effects and detailed mechanism of action of shikonin through its regulatory effects on the expression of proinflammatory cytokines and chemokines in dendritic cells. Understanding the therapeutic effects of shikonin on AD treatment can provide hope for new drug development with fewer side effects and a lower cost than those of currently available drugs.

## 2. Materials and Methods

### 2.1. Participants

A total of 10 patients with AD and 7 healthy controls were enrolled in this study. Patients with AD were included if they met the criteria of Hanifin and Rajka [19] including pruritus, typical morphology and distribution, chronic or chronically relapsing dermatitis, and personal or family history of atopy. All patients with AD were sensitive to HDM, and HDM-specific IgE was measured by using the Bio IC system (Agnitio, Taiwan). All participants were selected from the clinic of the Dermatology Department of Taichung Veterans General Hospital.

### 2.2. Ethics Statement

All participants provided written informed consent. The protocols and all research involving human participants were approved by the Institutional Review Board of Taichung Veterans General Hospital (TCVGH-CF-13038A).

### 2.3. Chemicals

Purified recombinant protein Der p 2 was purchased from Indoor Biotechnologies (Charlottesville, Virginia, USA). Shikonin was purchased from Sigma-Aldrich (St. Louis, MO, USA).

### 2.4. Cell Culture

For peripheral blood mononuclear cell (PBMC) culture, 16 mL of blood was collected from the patients in sodium citrate tubes (Vacutainer® CPT™, BD, USA), and PBMCs were purified through centrifugation over a density gradient. CD14^+^ cells were negatively selected using an MACS microbead column according to the manufacturer's protocol (Miltenyi Biotec, USA). Cells were cultured in RPMI-1640 supplemented with 10% fetal bovine serum, 1% penicillin/streptomycin, 25 mM HEPES, and 2 mM L-glutamine. CD14^+^ cells were further differentiated to obtain dendritic cells by stimulation with 50 ng/mL of recombinant granulocyte macrophage colony-stimulating factor (GM-CSF; PeproTech, USA) and 10 ng/mL of recombinant human IL-4 (PeproTech, USA) in a 6-well plate at 37°C and 5% CO_2_ for 7 days. Nonadherent dendritic cells were removed using phosphate-buffered saline, and the remaining dendritic cells were collected for subsequent experiments. A total of 6 × 10^5^ cells/mL were cultured in a 12-well plate and treated for 24 hours with the following: 1.5 *μ*g/mL of Der p 2, 1.5 *μ*g/mL of Der p 2, and 50 ng/mL shikonin, or 1.5 *μ*g/mL of Der p 2 and 10^−7^ M dexamethasone. The supernatant was collected for the subsequent measurement.

Cell viability was determined using the trypan blue dye exclusion assay.

Dendritic cells were treated with 0.016, 0.05, and 0.15 *μ*g/mL of shikonin for 24 hours. After treatment, cells were centrifuged and suspended in culture medium and mixed with trypan blue (Biological Industries, Kibbutz Beit Haemek, Israel) at a ratio of 1 : 1. A hemocytometer was used to count cells. Cell viability was calculated as the number of viable cells divided by the total number of cells [20].

### 2.5. Multiplex Assay

For measuring cytokine and chemokine levels, culture supernatants were collected and the concentrations of IL-1*β*, IL-1ra, IL-2, IL-4, IL-5, IL-6, IL-7, IL-8, IL-9, IL-10, IL-12, IL-13, IL-15, and IL-17A; eotaxin; basic fibroblast growth factor; granulocyte colony-stimulating factor; GM-CSF; interferon-*γ*; interferon *γ*-induced protein-10; monocyte chemoattractant protein (MCP)-1(MCAF); macrophage inflammatory protein (MIP)-1*α* and MIP-1*β*; platelet-derived growth factor-BB; Chemokine (C-C motif) ligand 5 (RANTES); tumor necrosis factor (TNF)-*α*; and vascular endothelial growth factor were determined using a protein multiplex immunoassay system (Bio-Plex Cytokine Array System, Bio-Rad Laboratories, Hercules, CA, USA).

### 2.6. Statistical Analyses

All statistical analyses were performed using SPSS, version 22 (IBM, Chicago, IL) by the Biostatistics Task Force of Taichung Veterans General Hospital, Taichung, Taiwan. The Wilcoxon signed-rank test was used, and data are presented as the mean ± standard deviation (SD); *p* < 0.05 was considered statistically significant.

## 3. Results

### 3.1. Comparison of Cytokine Profiles between Patients with AD and Healthy Controls

In this study, 10 patients with AD and 7 healthy controls were enrolled. Six of the 10 patients received melone, and all the 10 patients received topical steroid and oral histamine for control AD. Demographic characteristics, namely sex, age, asthma, allergic rhinitis status, EASI score, total IgE concentration, mite-specific IgE concentration, steroid usage, and TYO usage, were recorded (Tables 1 and 2). Dendritic cells were isolated and cultured for 24 hours. Subsequently, supernatants were collected for the cytokine profile analysis. The focus was mainly on innate cytokine and chemokine expression and cytokine and chemokine expression related to Th2 cells, Th17 cells, and macrophages.

Without Der p 2 induction, IL-1ra was significantly higher in the AD patients than in the controls (*p* < 0.05). Th2-dominant cytokine expression (IL-4, IL-9, and IL-13) levels were higher in the AD patients than in the controls (*p* > 0.05). Macrophage-related cytokine expression (MIP-1*α* and MIP-1*β*) levels were higher in the AD patients than in the controls (*p* > 0.05). IL-7 is produced by keratinocytes, dendritic cells, neurons, and other epithelial cells. The IL-7 level was higher in the AD patients than in the controls (*p* > 0.05). The levels of IL-17A and RANTES were higher in the controls than in the AD patients (*p* > 0.05). The aforementioned effect might have been observed because some patients received oral prednisolone (Table 3).

### 3.2. Proinflammatory Cytokine Expression Reduced in the Patients after Treatment with Shikonin

**Figure 2:**
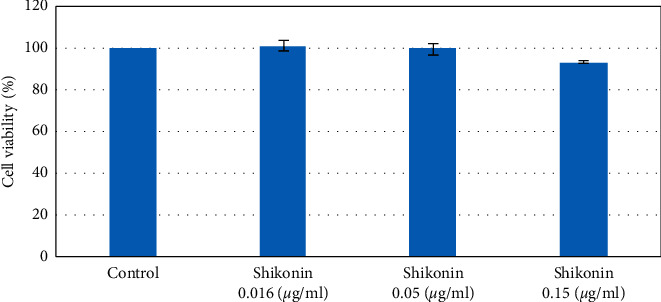
Cytotoxic effect of shikonin on dendritic cells isolated from patients with AD. Dendritic cells were treated with 0.016, 0.05, and 0.15 *μ*g/mL of shikonin for 24 hours. After treatment, cell viability was assessed using the trypan blue exclusion assay.

In total, 27 cytokines were analyzed from the supernatants of dendritic cell cultures, and we found that Der p 2 significantly induced the expression of IL-1*β*, IL-1ra, IL-6, IL-8, IL-9, IL-15, and IL-17; eotaxin; MCP-1; MIP-1*α*; MIP-1*β*; RANTES; and TNF-*α* compared with basal levels (data not shown). Before determining the effects of shikonin on proinflammatory cytokine production, the toxicity of shikonin on dendritic cells was measured using the trypan blue exclusion assay. Results showed that no toxic effects were detected after dendritic cells were cocultured with 0.016, 0.05, and 0.15 *μ*g/mL of shikonin for 24 hours (Figure 2).

**Figure 3:**
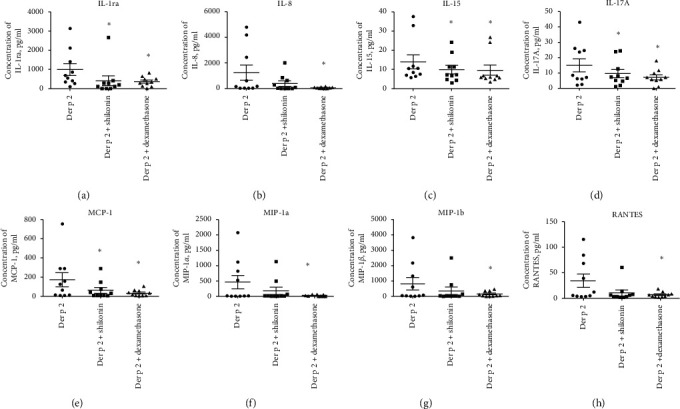
Effects of shikonin on inhibiting proinflammatory cytokine and chemokine expression. Dendritic cells isolated from patients with AD and incubated with 1.5 g/mL of Der p 2; 1.5 g/mL of Der p 2 and 0.05 g/mL of shikonin; and 1.5 g/mL of Der p 2 and 10−7 M dexamethasone for 24 hours. Culture supernatants were collected, and cytokine and chemokine expression were measured using the multiplex assay system. (a) IL-1ra, (b) IL-8, (c) IL-15, (d) IL-17A, (e) MCP-1, (f) MIP-1a, (g) MIP-1b, (h) RANTES.

Results also showed that in the AD patients, the levels of IL-1ra, IL-15, IL-17A, and MCP-1 were significantly lower in the shikonin-treated group than in the Der p 2-stimulated group (*n* = 10). The levels of IL-8, MIP-1*α*, MIP-1*β*, and RANTES were also lower in the shikonin-treated group than in the Der p 2-stimulated group; however, the difference was not significant. The expression levels of all cytokines were significantly lower in the dexamethasone-treated group than in the Der p 2-stimulated group (Figure 3). According to these results, 7 of the 10 patients were sensitive to shikonin treatment (patient number 4–10). By contrast, dendritic cells isolated from the remaining three patients exhibited higher levels of inflammatory cytokines after shikonin treatment than before treatment (patient number 1–3). We confirmed that no differences were observed in sex; EASI score; drug history; and combined other atopic disease, such as asthma or rhinitis, between the shikonin-sensitive patients and the other three patients (data not shown). Based on the effect of shikonin, three groups were distinguished (worse than prednisolone N1-3, better than or equal to prednisolone N4, and excellent response over prednisolone N5-10). We further analyzed the data in the excellent-response group, and results showed that Th2, Th17, and macrophage-dominant cytokines (namely, IL-6, IL-9, IL-17, MCP-1, MIP-1*α*, MIP-1*β*, and RANTES) were significantly downregulated after shikonin treatment (Table 4). Dexamethasone mainly inhibited Der p 2-induced proinflammatory cytokine expression (Table 5). Moreover, we compared the levels of proinflammatory cytokines between shikonin- and dexamethasone-treated cells, and results showed that expression levels of IL-9, MIP-1*β*, and RANTES were significantly lower in the shikonin group than in the dexamethasone group (Table 6). These data indicate that shikonin treatment can inhibit Der p 2-induced proinflammatory cytokine expression and exhibit better results than steroids in some patients with AD.

## 4. Discussion

Numerous experiments and clinical trials are in progress for developing new drugs to treat AD. However, only dupilumab, a monoclonal antibody against IL-4RA, has been approved by the United States FDA in recent years. The high cost and high risk of life-threatening infection cause concern and discontent among patients with AD. Partial AD patients still showed poor response to biologics. Thus, to discover a new inexpensive drug with fewer side effects becomes necessary. In this study, our data revealed that shikonin reduced Der p 2-induced Th2-, Th17-, and macrophage-related cytokine expression in dendritic cells isolated from HDM-allergic patients with AD.

IL-1 receptors mediated multiple proinflammatory responses, including enhancing expression of cytokines related to Th2 and Th17 as well as inducing proinflammatory cytokine production [21–23]. IL-1 receptors can conjugate with different cytokines such as IL-1*β*, IL-18, and IL-33 [24]. After activation by the agonist, the signaling pathway initiated transcription of proinflammatory cytokines, leading to inflammatory responses [25]. In normal homeostasis, when cells receive excessive inflammatory stimuli, they start creating negative feedback loops to offset inflammation. In the case of a skin disorder, activated keratinocytes produce a neuropeptide to enhance IL-10 expression for creating negative feedback signals for modulating inflammatory responses in the skin [26]. In the present study, we found that the basal level of IL-1ra in patients with AD was significantly higher than that in the controls. IL-1ra has been reported to bind to IL-1RI and block the activity of IL-1*α* or IL-1*β* [27]. This result suggested that IL-1ra served as a negative regulator, and its increased expression in patients with AD was expected to inhibit inflammatory responses.

**Figure 4:**
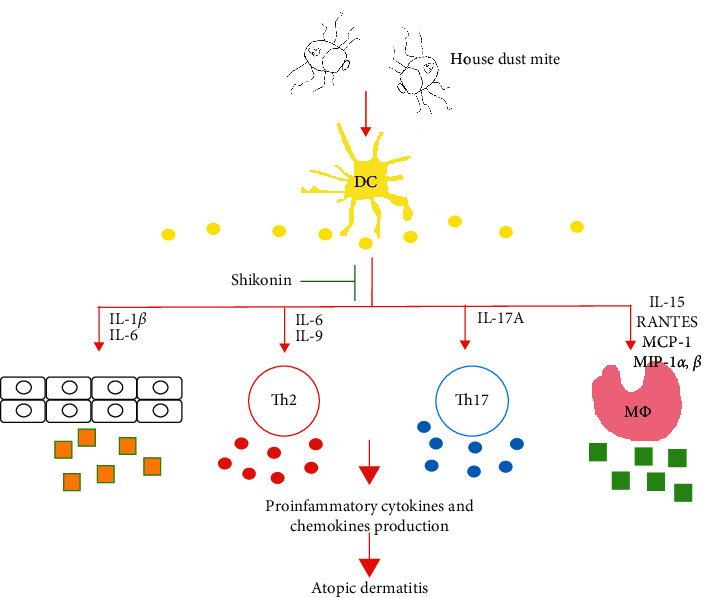
Schematic diagram of the proposed action of shikonin om Der p 2-induced dendritic cell activation and proinflammatory cytokine production.

Proinflammatory cytokines secreted by Th2 cells, Th17 cells, and macrophages play a major role in AD pathogenesis. The levels of IL-4, IL-5, and IL-13 have been reported to be higher in the skin of patients with AD and upregulated IgE levels were correlated with higher expression of IL-5 and IL-13 in patients with AD [28]. Overexpression of Th2 cell-related cytokines was reported to cause epidermal thickening, inflammation, eosinophilia, pruritus and magnify the symptoms of AD [29]. By contrast, Th17 overactivation was observed in Asian patients with AD and early-onset pediatric patients with AD [30]. Th17 cells express proinflammatory cytokines, such as IL-17A and IL-22, and their differentiation is regulated by IL-6, TGF-*β*, and IL-1*β* [31]. IL-17 can stimulate epithelial cells and fibroblasts to produce cytokines and chemokines, such as IL-6 and IL-8, attract additional immune cells, infiltrate into skin lesions, and cause cutaneous remodeling in AD. Overexpression of IL-17 has been reported to enhance fibrosis and chronic dermatitis and stimulate eosinophils to secrete other chemokines, which causes exacerbation of dermatitis [32, 33]. In our data, shikonin and dexamethasone reduced expression of Th17-related cytokines, IL-1*β*, IL-6, and IL-17 and that of Th2-related cytokines, IL-6 and IL-9, which were secreted by dendritic cells. By contrast, minimal levels of IL-4, IL-5, and IL13 were observed, which was consistent with previous reports in which no secretion by dendritic cells was observed [34]. Shikonin and dexamethasone attenuated IL-6 and IL-9 secretion by dendritic cells, which can modulate the Th2 pathway and control AD. Shikonin downregulated IL-9 more effectively than dexamethasone in 6 of the 10 patients. Tracing back our clinical data, the 10 patients responded satisfactorily to steroids; this result was comparable to those of our dendritic cytokine and chemokine tests. Two patients (patient 7 and 8) were treated using TYO; they exhibited a highly positive response to clinical treatment. Both of them responded more satisfactorily to TYO than to topical steroids subjectively and objectively. Patient 3 also used TYO, but his condition deteriorated. The clinical response of Patient 3 to TYO was consistent with the upregulating effects of shikonin. Dendritic cells activated by Der p 2-related cytokines, such as MCP-1, MIP-1*α*, and MIP-1*β*, can stimulate macrophages to secrete increased amounts of cytokines and chemokines to attract additional immune cells to infiltrate skin lesions and form a positive loop, leading to severe dermatitis [35, 36]. Furthermore, the effects of IL-15 secretion by activated macrophages and dendritic cells remain controversial. Decreased IL-15 expression has been reported to contribute to the pathogenesis of AD by enhancing IgE production [37]. By contrast, IL-15 could enhance T-cell recruitment and survival in the skin [38]. Our results showed that shikonin treatment could significantly reduce Der p 2-induced MCP-1, MIP-1*α*, MIP-1*β*, and IL-15 expression in dendritic cells isolated from HDM-allergic patients with AD. It suggested that shikonin could attenuate chemokine orchestration. RANTES is a potent chemoattractant for eosinophils, monocytes, basophils, and lymphocytes. Serum RANTES levels are correlated with IgE levels, total eosinophil counts, and lactate dehydrogenase levels [39]. It plays a major role in eosinophil infiltration and augmentation in disease severity [40]. In our study, RANTES was secreted by Der p 2-activated dendritic cells and downregulated by shikonin. Dendritic cells doubtlessly play an important role in connecting the innate and adaptive immune systems [41]. In this study, dendritic cells were the focus for investigating the efficacy of shikonin on inhibiting Der p 2-induced proinflammatory cytokine and chemokine expression. In the present study, we demonstrated that shikonin reduced the expression of the proinflammatory cytokines IL-6 and IL-17A and production of the macrophage-related cytokines and chemokines MCP-1, MIP-1*α*, and MIP-1*β*. Moreover, shikonin inhibited production of the proinflammatory cytokines IL-9, MIP-1*β*, and RANTES more effectively than dexamethasone. In the recent year, CCL17 and CCL22 have been reported that played an important role in Th2 cytokines induced AD [42]. CCL17 and CCL22 could be secreted by dendritic cells not only developed the inflammatory responses but also became pivotal regulator of the AD pathogenesis. These chemokines expression regulated by shikonin in our model will be investigated in the future. The schematic diagram of how shikonin ameliorates dendritic cell activation and treats AD is shown in Figure 4.

According to *in vitro* and clinical data, shikonin can regulate the overactivated immune response in patients with AD and act as an alternative medication to treat AD with lower risks and fewer side effects than the current medications.

The effects of shikonin on inhibiting Der p 2-induced proinflammatory cytokine expression and dermatitis in patients with AD have been demonstrated in this study. However, this study has some limitations. First, dendritic cells were isolated from only 10 patients with AD; however, the expression of proinflammatory cytokines could not be inhibited in three patients and only one patient exhibited trace inhibition effects after cells were cocultured with Der p 2 and shikonin (data not shown). Interindividual variability in response to the same drug could be due to physiological variables, pathological factors, genetic variation of key enzymes of receptors, and interaction with other drugs [43]. This variability also revealed the importance of precision medical treatment for reducing AD, and the methodology we used in this study could be used as a platform for screening patients with AD with a satisfactory response to the shikonin treatment. Second, in this study, only 10 patients with AD were enrolled to evaluate the effects of shikonin on inhibiting proinflammatory cytokine production. Dendritic cell samples isolated from a large number of patients with AD and more dendritic cells expressed chemokines such as CCL17 and CCL22 which were related to AD pathogenesis should be investigated in future studies. Furthermore, clinical trials should be used to evaluate the direct effects of shikonin on ameliorating dermatitis of patients with AD. Third, the pathogenesis of AD is highly complicated, and different immune cells, skin microorganisms, and skin barriers are involved. Therefore, the effects of shikonin on other immune cells and keratinocytes as well as modulating the composition of microorganisms should be validated in future studies.

## 5. Conclusion

In conclusion, to our knowledge, this is the first report on shikonin-mediated inhibition of expression of proinflammatory cytokines by dendritic cells in patients with AD. Through this study, we provided a novel screening platform for individual treatment and evidence that shikonin is a promising potential therapy for AD.

## Figures and Tables

**Table 1:** Demographic and clinical characteristics.

	Healthy subject (*n* = 7)	AD patients (*n* = 10)
Sex		
Male	5 (71.4)	6 (60%)
Female	2 (28.6)	4 (40%)
Age (yrs)	41.1 ± 17.5	29.8 ± 11.1
EASI score	N. A	15.3 ± 11.7
IgE (IU/mL)	N. A	3335.2 ± 1779.2
Mite-specific IgE	N. A	10 (100%)
Topical steroid treatment	0 (0%)	10 (100%)
Systemic steroid treatment	0 (0%)	6 (60%)
Asthma	0 (0%)	5 (50%)
Rhinitis	2 (28.6)	6 (60%)
HBsAg	0 (0%)	0 (0%)
Anti-HCV	0 (0%)	1 (10%)
Autoimmune disease	0 (0%)	0 (0%)
Diabetes mellitus	1 (14.2%)	0 (0%)
Hypertension	1 (14.2%)	0 (0%)

Values are expressed as the number (percentage) or mean ± standard deviation.

**Table 2:** Demographic and clinical characteristics of patients with atopic dermatitis.

	Age	EASI score	Topical steroid treatment	Systemic steroid treatment	IgE kU/l^#^	Asthma	Rhinitis	HBsAg	Anti-HCV	Autoimmune disease	Diabetes mellitus	Hypertension	Tzu-Yun ointment
Patient 1	25	26	O	o	>5000	x	X	x	x	X	x	x	x
Patient 2	21	5	O	x	1801	x	X	x	x	X	x	x	x
Patient 3	22	20	O	o	4451	x	X	x	x	X	x	x	o
Patient 4	39	10	O	x	>5000	o	O	x	x	X	x	x	x
Patient 5	28	26	O	o	1129	o	O	x	x	X	x	x	x
Patient 6	58	2	O	x	550	o	O	x	x	X	x	x	x
Patient 7	26	28	O	o	>5000	x	O	x	x	X	x	x	o
Patient 8	23	30	O	o	>5000	o	O	x	o	X	x	x	o
Patient 9	29	4	O	x	2262	o	O	x	x	X	x	x	x
Patient 10	27	25	O	o	3159	x	X	x	x	X	x	x	x

^#^The detection limit of IgE is 5000 kU/L.

**Table 3:** Basal level of the cytokine profile from healthy controls and patients with atopic dermatitis.

	Healthy subject (*n* = 7)		AD patient (*n* = 10)		*p* value
	Mean	SD	Mean	SD	
IL-1*β*	0.4	±0.4	1.1	±1.6	0.732
IL-1ra	312.3	±571.7	680.5	±612.1	0.040^*∗*^
IL-2	1.9	±1.5	2.2	±1.3	0.557
IL-4	0.3	±0.2	0.5	±0.4	0.435
IL-5	0.8	±1.2	0.8	±1.2	0.913
IL-6	1.5	±1.2	1.2	±0.7	0.733
IL-7	0.7	±1.9	3.2	±4.8	0.101
IL-8	20.7	±5.2	70.2	±87.4	0.845
IL-9	7.0	±5.5	12.3	±12.2	0.526
IL-10	2.0	±1.0	2.7	±1.7	0.435
IL-12 (*p*70)	3.0	±2.1	2.1	±1.8	0.464
IL-13	0.3	±0.4	1.5	±2.1	0.157
IL-15	9.6	±8.9	10.9	±9.2	0.696
IL-17A	11.5	±5.4	7.3	±3.8	0.051
Eotaxin	2.7	±1.6	3.0	±2.3	0.884
Basic FGF	27.7	±11.4	30.8	±19.6	0.626
G-CSF	11.4	±19.8	12.4	±9.3	0.187
GM-CSF	92.3	±44.4	90.7	±65.0	0.626
IFN-*γ*	4.3	±6.4	5.9	±3.7	0.239
IP-10	1.4	±2.7	1.2	±2.0	0.819
MCP-1	122.7	±76.4	66.8	±63.8	0.079
MIP-1*α*	15.0	±17.1	25.2	±29.7	0.558
MIP-1*β*	45.9	±44.2	81.8	±98.8	0.922
PDGF-BB	3.7	±2.1	5.9	±5.9	0.695
RANTES	17.3	±35.6	5.5	±2.5	0.406
TNF-*α*	6.3	±10.6	2.6	±1.8	0.806
VEGF	28.0	±35.6	19.7	±23.3	0.625

Data are expressed as the mean ± standard deviation. Data were analyzed using the Wilcoxon signed-rank test.

**Table 4:** Proinflammatory cytokine levels of the Der p 2 group and shikonin-treated group (*n* = 6).

	Der p 2		Shikonin		*p* value
	Mean	SD	Mean	SD	
IL-4	0.5	±0.4	0.3	±0.4	0.138
IL-5	0.7	±1.7	0.9	±1.1	0.715
IL-6	3.3	±4.1	0.7	±0.7	0.043^*∗*^
IL-9	21.6	±22.6	5.3	±5.3	0.028^*∗*^
IL-13	1.3	±2.2	1.2	±1.5	0.893
IL-17A	12.1	±9.6	5.5	±2.6	0.027^*∗*^
MCP-1	91.0	±112.9	15.6	±14.5	0.028^*∗*^
MIP-1*α*	330.6	±505.5	9.9	±6.7	0.046^*∗*^
MIP-1*β*	610.2	±919.4	6.9	±7.2	0.028^*∗*^
RANTES	29.8	±36.4	3.6	±2.0	0.028^*∗*^

Data are expressed as the mean ± standard deviation (SD). Data were analyzed using the Wilcoxon signed-rank test.

**Table 5:** Proinflammatory cytokine level of the Der p 2 group and dexamethasone-treated group (*n* = 6).

	Der p 2		Dexamethasone		*p* value
	Mean	SD	Mean	SD	
IL-4	0.5	±0.4	0.3	±0.1	0.068
IL-5	0.7	±1.7	0.4	±1.1	0.317
IL-6	3.3	±4.1	0.7	±0.7	0.027^*∗*^
IL-9	21.6	±22.6	21.9	±17.3	0.463
IL-13	1.3	±2.2	3.6	±4.5	0.080
IL-17A	12.1	±9.6	6.7	±4.1	0.046^*∗*^
MCP-1	91.0	±112.9	32.5	±37.9	0.028^*∗*^
MIP-1*α*	330.6	±505.5	21.8	±25.2	0.028^*∗*^
MIP-1*β*	610.2	±919.4	147.7	±173.9	0.116
RANTES	29.8	±36.4	8.2	±6.0	0.028^*∗*^

Data are expressed as the mean ± standard deviation (SD). Data were analyzed using the Wilcoxon signed-rank test.

**Table 6:** Proinflammatory cytokine levels of the shikonin-treated group and dexamethasone-treated group (*n* = 6).

	Shikonin		Dexamethasone		*p* value
	Mean	SD	Mean	SD	
IL-4	0.33	±0.36	0.26	±0.14	0.465
IL-5	0.90	±1.09	0.44	±1.08	0.068
IL-6	0.74	±0.73	0.75	±0.66	1.000
IL-9	5.26	±5.31	21.90	±17.35	0.046^*∗*^
IL-13	1.23	±1.46	3.63	±4.52	0.075
IL-17A	5.53	±2.56	6.73	±4.12	0.249
MCP-1	15.58	±14.45	32.52	±37.89	0.116
MIP-1*α*	9.91	±6.72	21.77	±25.23	0.463
MIP-1*β*	6.92	±7.17	147.70	±173.91	0.046^*∗*^
RANTES	3.60	±2.03	8.23	±5.97	0.043^*∗*^

Data are expressed as the mean ± standard deviation (SD). Data were analyzed using the Wilcoxon signed-rank test.

## Data Availability

The data used to support the findings of this study are available from the corresponding author upon request.
